# Control of translational activation by PIM kinase in activated B-cell diffuse large B-cell lymphoma confers sensitivity to inhibition by PIM447

**DOI:** 10.18632/oncotarget.11457

**Published:** 2016-08-20

**Authors:** Tara L. Peters, Lingxiao Li, Ana A. Tula-Sanchez, Praechompoo Pongtornpipat, Jonathan H. Schatz

**Affiliations:** ^1^ Sheila and David Fuente Graduate Program in Cancer Biology, Division of Hematology-Oncology, Sylvester Comprehensive Cancer Center, University of Miami Miller School of Medicine, Miami, FL, USA; ^2^ Department of Medicine, Division of Hematology-Oncology, Sylvester Comprehensive Cancer Center, University of Miami Miller School of Medicine, Miami, FL, USA; ^3^ Department of Molecular and Cellular Biology, University of Arizona, Tucson, AZ, USA; ^4^ Bio5 Institute, University of Arizona, Tucson, AZ, USA

**Keywords:** ABC-DLBCL, PIM kinase, cap-dependent translation, B-cell receptor signaling, lymphomagenesis

## Abstract

The PIM family kinases promote growth and survival of tumor cells and are expressed in a wide variety of human cancers. Their potential as therapeutic targets, however, is complicated by overlapping activities with multiple other pathways and remains poorly defined in most clinical scenarios. Here we explore activity of the new pan-PIM inhibitor PIM447 in a variety of lymphoid-derived tumors. We find strong activity in cell lines derived from the activated B-cell subtype of diffuse large B-cell lymphoma (ABC-DLBCL). Sensitive lines show lost activation of the mTORC1 signaling complex and subsequent lost activation of cap-dependent protein translation. In addition, we characterize recurrent *PIM1* protein-coding mutations found in DLBCL clinical samples and find most preserve the wild-type protein's ability to protect cells from apoptosis but do not bypass activity of PIM447. Pan-PIM inhibition therefore may have an important role to play in the therapy of selected ABC-DLBCL cases.

## INTRODUCTION

The proviral insertion in murine leukemia virus (PIM) family proteins are serine/threonine kinases that promote growth and survival in multiple cell types [1–3]. More than 30 years ago, *PIM1* emerged from a viral-integration screen designed to identify genes whose expression synergized with c-MYC to promote oncogenesis [4]. *PIM2* and *PIM3* were found over the decade that followed through ability to rescue *PIM1* deletion and structural homology [5, 6]. With largely overlapping substrates, the PIMs are found expressed in cells throughout hematopoietic cell lineages [7, 8] and in multiple other cell types, including vascular smooth muscle [9], cardiomyocytes [10], and breast [11]. They are constitutively active kinases regulated through expression and rapid turnover downstream of growth factor signaling [12, 13]. Their pro-growth and survival endpoints, direct and indirect, include activation of cap-dependent protein translation initiation, progression through the cell cycle, and inhibition of apoptosis, among others [1–3].

Elevated expression of all three family members is found in a variety of human malignancies, including non-Hodgkin lymphomas [14–17], acute leukemias [18, 19], multiple myeloma [20–22], and prostate [23], hepatocellular [24], and colon [25] cancers, among others. PIM activity appears non-essential in both adult homeostasis and development, as even *Pim1/Pim2/Pim3* triple knockout mice are viable, though demonstrating decreased body size and diminished immune effector-cell activation [7]. Further strengthening a case for therapeutic inhibition, the PIM kinase domain has an unusual 3D structure, containing an atypical proline hinge region, fueling extensive efforts to develop potent and specific inhibitors [13, 26, 27]. Early efforts focused on PIM1 specifically, and a compound with potent PIM1 inhibitory activity, SGI-1776 [28], entered phase 1 clinical evaluation for patients with relapsed or refractory prostate cancer or lymphoma in 2008. This trial ended early due to off-target cardiac toxicity, and simultaneously most pharma-industry efforts shifted to pan-PIM inhibition. The multiple functional redundancies of the three family members in preclinical systems combined with their common expression provides immediate potential source of resistance/treatment failure from inhibiting any one family member alone.

PIM447 (formerly LGH447) is an orally available clinical pan-PIM kinase inhibitor structurally related to the potent tool compound LGB321 and currently in phase 1 clinical evaluation in multiple myeloma [29]. Myeloma shows common expression of PIM2 in particular [20–22], and early results announced at international meetings suggest promising clinical activity in heavily pretreated relapsed populations (see discussion). Here we assessed the activity of PIM447 in cell lines derived from a variety of additional lymphoid malignancies. Results led us to focus further studies on the poor-prognosis activated B-cell-derived subtype of diffuse large B-cell lymphoma (ABC-DLBCL). We find high dependence on PIM in most ABC-DLBCL lines to preserve activation of cap-dependent protein translation. In addition, we undertake functional analysis of PIM1 protein-coding mutations, which are commonly found in DLBCL clinical samples.

## RESULTS

### Pan-PIM kinase inhibition in ABC-DLBCL

**Figure 1:**
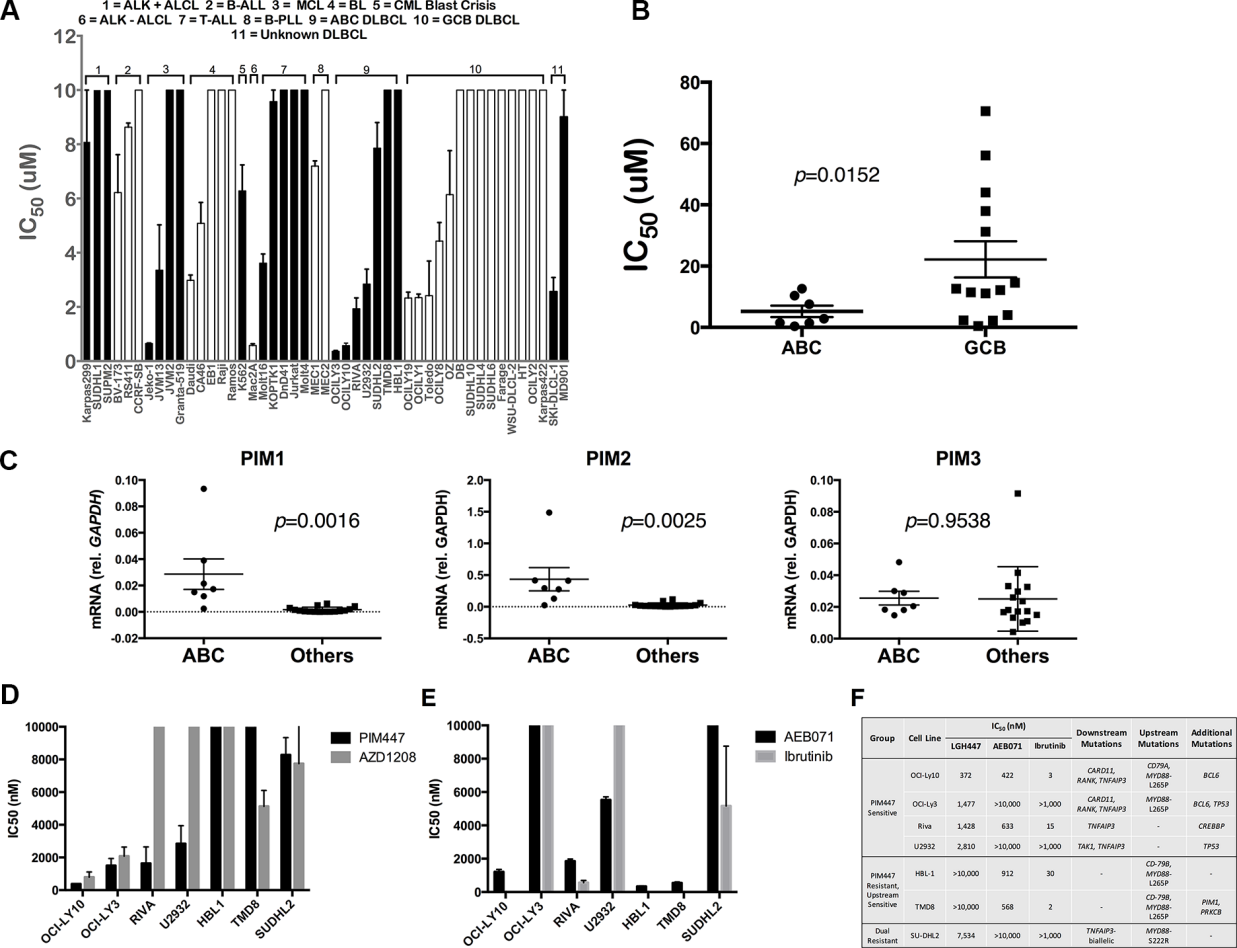
Pan-PIM Inhibition with PIM447 in non-myeloma lymphoid tumor cell lines (**A**) IC_50_ values of cell lines to PIM447. IC_50_ values determined from luminescent viability with the Cell Titer Glo reagent. Indicated cell lines were treated with serial dilutions of PIM447 for 72 hours. Shown is the mean of four replicates ± SEM. (**B**) IC_50_ values of ABC and GCB DLBCL cell lines, compared with two-tailed *T* test. (**C**) mRNA expression of *PIM1*, *PIM2* and *PIM3* in ABC-derived vs. other DLBCL cell lines, compared with two-tailed *T* test. qRT-PCR was performed on cDNA isolated from each cell line. 2^−ΔΔCT^ normalized to GAPDH (**D**) IC_50_ values calculated from cell viability assays of indicated cell lines, with the top concentration for PIM447 and AZD1208 being 10 μM. Shown is the mean of four replicates ± SEM. (**E**) IC_50_ values calculated from cell viability assays of indicated cell lines, with the top concentration for AEB071 being 10 μM and 1 μM for Ibrutinib. Shown is the mean of four replicates ± SEM. (**F**) ABC DLBCL cell lines, grouped based on drug sensitivity, with known somatic mutations.

Like LGB321, PIM447 has wide ranging activity in cell lines derived from various lymphoid malignancies (Figure 1A) [29]. In DLBCL, we note strongest activity against ABC-derived lines with 4/7 (57%), showing IC_50_ < 3 μM. Mean IC_50_ for ABC lines was significantly lower than GCB (*p* = 0.0152, Figure 1B). *PIM1* and *PIM2* previously were reported among genes whose expression is higher in ABC-DLBCL, helping to distinguish it from germinal-center B-cell (GCB) subtype [17]. Both may be turned on in expression by NF-kB activation, the pathogenic hallmark of the ABC subtype [30, 31]. Consistently, ABC lines show significantly higher *PIM1* (*p* = 0.0016) and *PIM2* (*p* = 0.0025) mRNA expression than the remaining DLBCL lines, while *PIM3* (*p* = 0.9538) was highly similar between the groups (Figure 1C). Within the ABC group, however, there was clearly differential dependence on PIM activity to maintain cellular viability, as 3/7 lines (43%) were insensitive to PIM447. We compared the activity of PIM447 to AZD1208 [19], the only other pan-PIM kinase inhibitor to have entered clinical evaluation, and found a similar pattern of activity against ABC-DLBCL cell lines, though AZD1208 was less active against Riva and U2932 and more active against TMD8 (Figure 1D). We also compared sensitivity of the ABC lines to two additional inhibitors: ibrutinib, which targets the Bruton's Tyrosine Kinase (BTK); and AEB071, which targets the beta isoform of Protein Kinase C (PKCβ) (Figure 1E). Interestingly, OCI-Ly3 and OCI-Ly10, the two lines with greatest sensitivity to both PIM447 and AZD1208, both have mutations in the coiled-coil domain of CARD11, promoting activation of NF-kB independent of upstream signaling [32, 33]. (We routinely confirm cell-line identity through short-tandem-repeat fingerprinting and independently confirmed the presence of all listed mutations in the cell lines we used for this report (data not shown).) Ibrutinib and AEB071 target BCR signaling upstream of CARD11, which participates in NF-kB activation as part of the CARD11/BCL10/MALT1 (CBM) complex. Figure 1F summarizes drug sensitivity results and known signaling-molecule mutations in the seven ABC lines, including *TNFAIP3* alterations in all four of the PIM447-sensitive lines [34–36]. *TNFAIP3* encodes the A20 protein, a downstream negative regulator of NF-kB activation. Taken together, these data suggest ABC tumors with activation of NF-kB signaling independent of upstream signaling are more likely to depend on PIM kinase activity. TMD8 and HBL1, by contrast, have no downstream mutations and instead activate BCR signaling upstream through both CD79B and MYD88-L265P mutations. Strikingly, both of these lines are completely insensitive to PIM447 but highly sensitive to both upstream inhibitors.

### Translational activation is inhibited upon PIM447 treatment in sensitive lines

**Figure 2:**
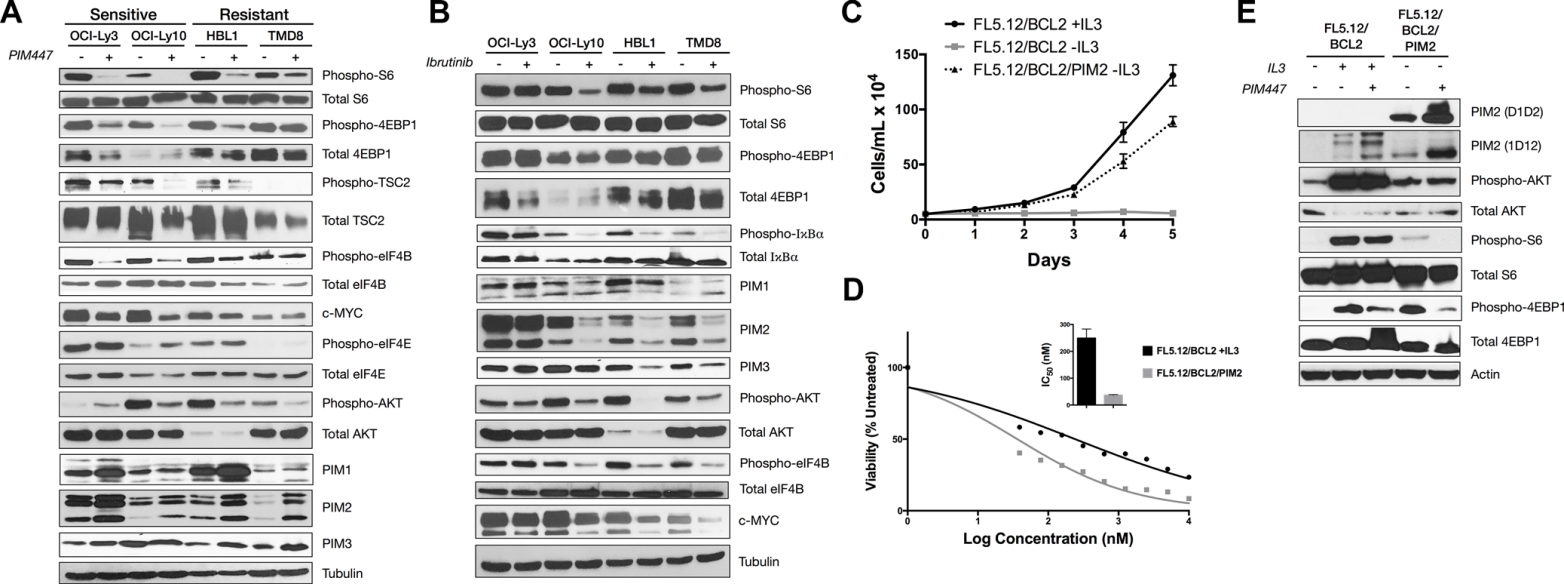
Loss of mTORC1 activation in PIM447-sensitive ABC-DLBCL lines and independent PIM2-driven system (**A**) PIM447-sensitive cell lines lose translational activation after treatment with PIM447. 5,000,000 cells were treated with 5 μM PIM447 for 16 hours and indicated proteins analyzed by western blot. (**B**) Upstream inhibitor sensitive cell lines lose translational activation when treated with Ibrutinib. 5,000,000 cells were treated with 1 μM Ibrutinib for 16 hours and protein was collected and analyzed as in A. (**C**) Cell proliferation of FL5.12/BCL2 cells in IL3 and PIM2-driven FL5.12/BCL2 cells. 50,000 cells were plated in media with and without IL3 as indicated and counted every day for 5 days. Mean ± SEM of three independent replicates. (**D**) Cell viability assay of FL5.12/BCL2 cells in PIM447. Viability of untransformed FL5.12/BCL2 cells in normal media and the transformed FL5.12/BCL2 PIM2 cells in media without cytokine was assessed after 72 hours using the Cell Titer Glo reagent and IC_50_s were calculated using non- linear curve fit regression. Shown is the mean of four replicates ± SEM. (**E**) 5,000,000 cells were treated with PIM447 for 16 hours in media containing IL3 as indicated and analyzed as described in A.

**Figure 3:**
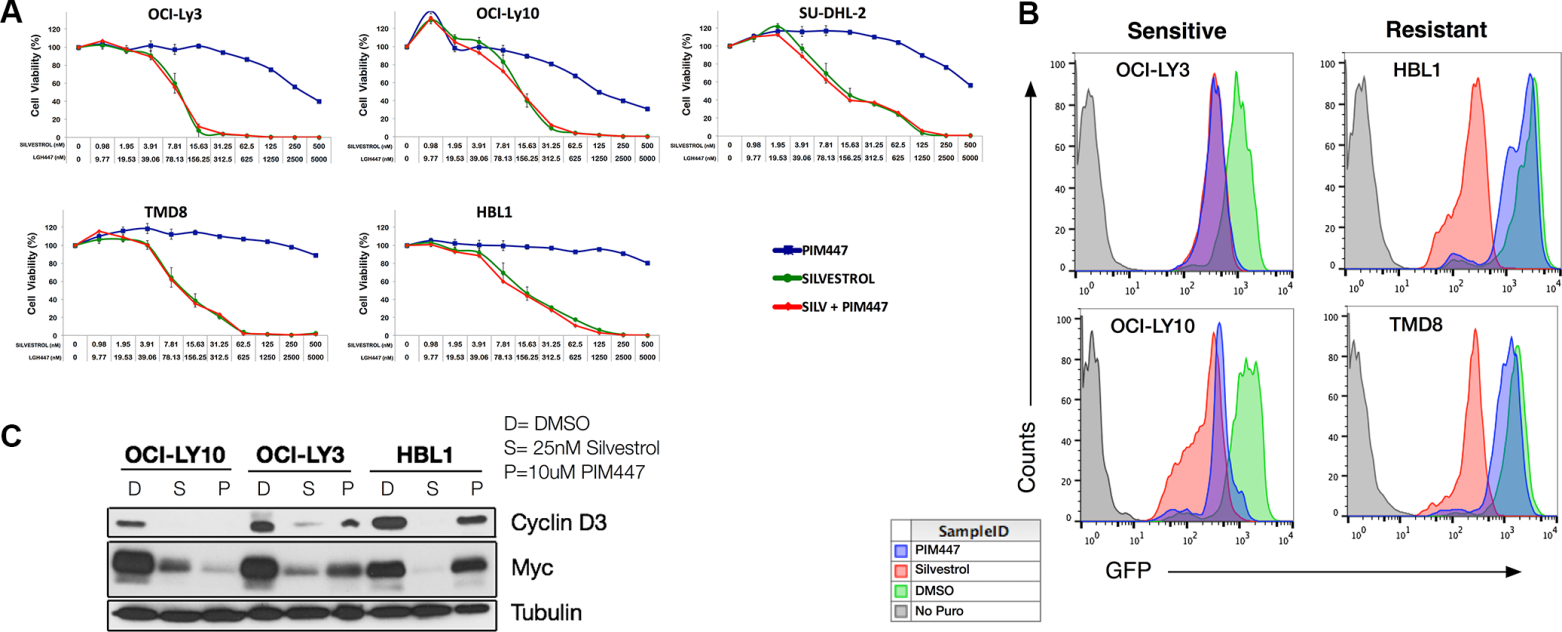
PIM447 treatment leads to a reduction in cap-dependent protein translation in sensitive cell lines (**A**) Cell viability assays with PIM447, Silvestrol, and the combination with the top concentration for PIM447 being 5 μM and 500 nM for Silvestrol. Cells were then incubated for 48 hours and viability was assessed using Cell Titer Glo reagent. Shown is the mean of four replicates ± SEM. (**B**) OPP incorporation after treatment with PIM447. 100,000 cells were plated and treated with either 10 μM PIM447, 100nM Silvestrol or vehicle and incubated for 24 hours. At 23 hours 50 μM OPP was added, then cells were harvested at 24 hours, and an Alexa Fluor 488 azide was attached to the OPP terminated proteins using the Click-iT Cell Reaction Buffer Kit and analyzed by FACS. (**C**) Translationally regulated proteins are lost after PIM447 treatment in sensitive cell lines. 5,000,000 cells were treated with 25 nM Silvestrol, 10 uM PIM447 or vehicle PIM447 for 16 hours and indicated proteins were analyzed by western blot.

Numerous downstream targets potentially are affected by PIM-kinase activity. Because of our prior work demonstrating the central importance of translational activation downstream of lymphoma survival signaling [15], we focused on activators of this process. mTORC1 activates translation through its downstream targets S6 Kinase and 4EBP1, both of which were potently inhibited in sensitive but substantially less so in resistant ABC-DLBCL lines (Figure 2A). PIM2 previously was shown to regulate mTORC1 activation through phosphorylation of its upstream negative regulator TSC2 at Ser-1798 [20]. Consistently, Figure 2A shows PIM447 treatment results in lost phosphorylation at this residue in sensitive lines only. PIM activity also activates translation through direct phosphorylation of eIF4B, a co-factor of the eIF4F translation-initiation complex [37]. Again, PIM447-sensitive lines show lost phosphorylation at this residue in response to the drug while resistant lines do not. These effects on translation result in decreased expression of the translationally regulated c-MYC oncoprotein (see also Figure 3C). As expected, PIM447 does not affect phosphorylation of eIF4E, which is regulated by MNK1/2 downstream of MAPK signaling [38]. Interestingly, AKT phosphorylation at S473 may be either activated or repressed due to PIM447 treatment, but there is no clear association with sensitivity or resistance. Finally, as has been observed by others, PIM kinase inhibition results in increases of PIM1 and PIM2 protein levels, likely due to inhibition of a previously described autophosphorylation-dependent degradation pathway [39]. We confirmed there was no increase of PIM mRNA in association with the increased protein levels during PIM447 exposure, consistent with increased protein stability rather than activation of expression (Figure S1). In sum, sensitivity to PIM447 in ABC-DLBCL associates with lost activation of cap-dependent translation and diminished expression of c-MYC. Increased stability of the PIM proteins themselves in response to pan-PIM inhibition, however, could be a limiting factor in efficacy of this therapeutic approach.

We next compared the effect of upstream inhibition with ibrutinib in the same four lines (Figure 2B), of which OCI-Ly3 is ibrutinib resistant, while the other three are sensitive (Figure 1D–1E). OCI-Ly3 shows no downstream signaling consequences from ibrutinib treatment, consistent with its insensitivity to the drug and activation of core survival signaling downstream through known *CARD11* and *TNFAIP3* mutations. The sensitive lines, by contrast, all show diminished phopho-S6 (although less effect on phospho-4EBP1) along with reduced phospho-IκBα, phospho-AKT S473, and PIM2 protein expression, which in turn associates with loss of phosph-eIF4B and reduced c-MYC. OCI-Ly10, in contrast to Ly3, retains some dependence on BTK for downstream activation despite being sensitive to PIM447, while TMD8 and HBL-1, which lack downstream mutations, are sensitive to upstream inhibition only.

**Figure 4:**
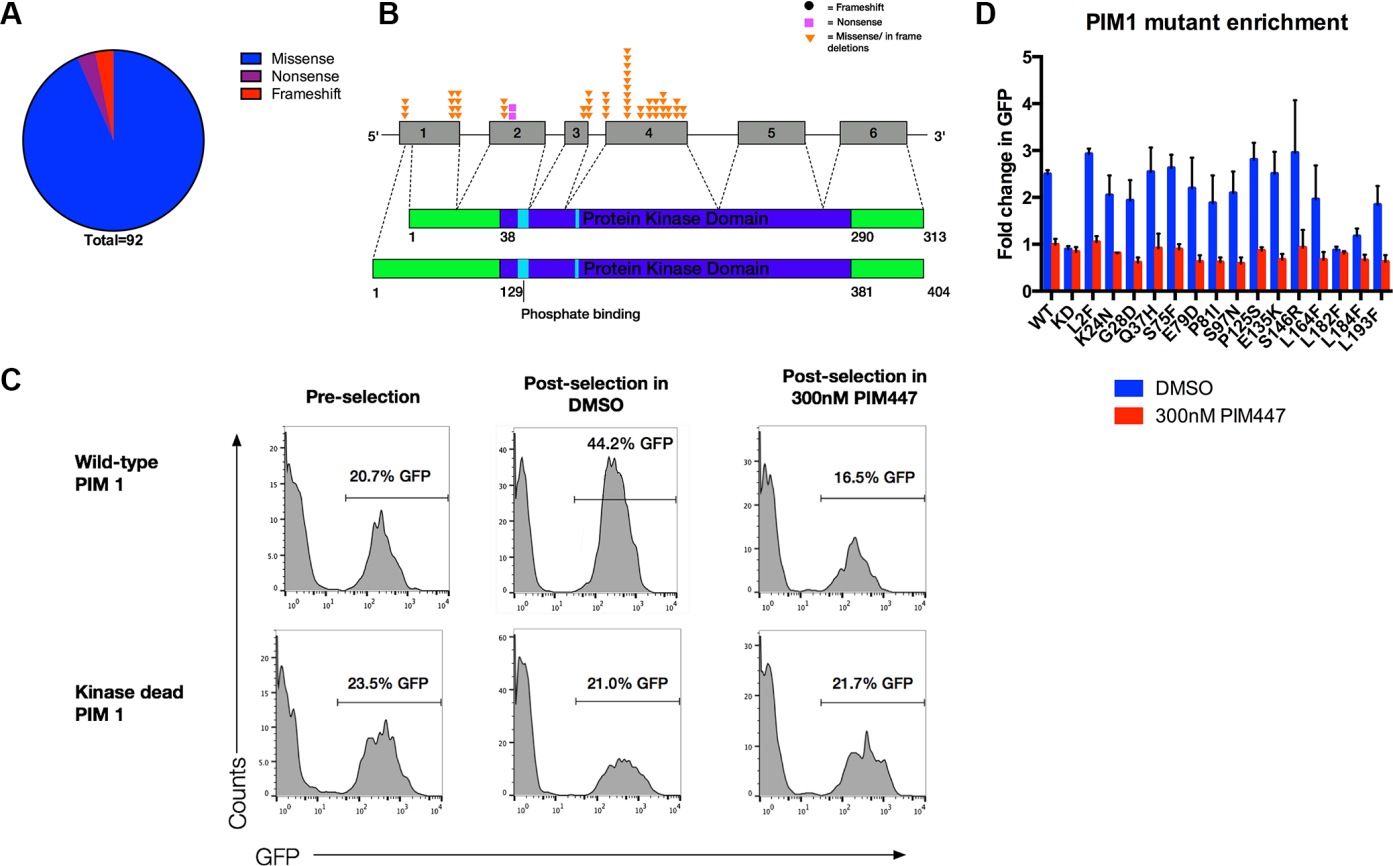
Majority of somatic mutations in PIM1 are missense mutations that preserve kinase function (**A**) There are a total of 92 somatic mutations in PIM1 identified in the literature, 86 of which are missense mutations, 3 are nonsense, and 3 are frame-shift. (**B**) Schematic showing the distribution of recurrent PIM1 somatic mutations. The short isoform of PIM1 is shown on the top while the long isoform is on bottom. (**C**) Wild-type, but not kinase dead, PIM1 is selected for upon IL3 withdrawal only when PIM function is not inhibited in FL5.12/BCL2 cells. 100,000 FL5.12/BCL2 cells were retrovirally infected with wild-type or kinase-dead PIM1 in pMIG vector, mixed to approximately 20% GFP positive cells, then washed out of IL3 for 24 hours and treated with either 300 nM PIM447 or vehicle, allowed to recover in IL3 with drug treatment and then analyzed by FACS. (**D**) Pooled enrichment results for three replicates, mean ± SEM, of wild-type, kinase-dead, and recurrent DLBCL *PIM1* mutants in FL5.12/BCL2 cells with and without PIM447. Competition assay was performed as described in C.

To create an independent PIM-dependent system, we introduced cDNA for the short form of human *PIM2* retrovirally to FL5.12 cells, which are IL3-dependent murine pro-B cells. *PIM2* expression in FL5.12 cells that constitutively express *BCL2* resulted in transformation to IL3 independence (Figure 2C). (*PIM2* introduction to FL5.12 cells without *BCL2* expression and *PIM1* introduction to FL5.12 cells with or without *BCL2* all resulted in strong selective advantage during IL3 withdrawal but did not permit transformation to IL3 independence (Figure 4C–4D and data not shown).) FL5.12/*BCL2*/*PIM2* cells are highly sensitive to PIM447 (IC_50_ 37 nM, Figure 2D). Increased PIM expression is a known pro-survival consequence of IL3 stimulation of FL5.12 cells [12, 40], and consistently FL5.12 *BCL2* cells growing in IL3 also show some sensitivity to PIM447 (IC_50_ 249 nM). Blotting with the α-PIM2 antibody clone D1D2, which recognizes human but not mouse PIM2, confirmed high expression of exogenous human PIM2 in the FL5.12/*BCL2*/*PIM2* cells, which was stabilized by PIM447 treatment (Figure 2E). The α-PIM2 antibody clone 1D12, which reacts to both human and mouse PIM2, shows endogenous PIM2 expression is IL3 dependent, as previously reported [12], and this too is stabilized by PIM447. These cells also completely lack mTORC1 activation without IL3, but this is only partially PIM447 sensitive associated with strong co-activation of AKT downstream of IL3 [12]. FL5.12/*BCL2*/*PIM2* cells, however, have no increased AKT activation over IL3-deprived FL5.12/*BCL2* cells but mTOR activation that is highly PIM447 sensitive. An independent PIM-transformed system, therefore, replicates the association between PIM inhibition and lost activation of mTORC1.

### PIM447-sensitive cells use PIM to maintain activation of translation

As shown above, several PIM targets that converge on activation of cap-dependent translational activation carried out by the eIF4F translation-initiation complex are inhibited by PIM447 in sensitive ABC-DLBCL cells. The plant-derived rocaglate silvestrol, meanwhile, is a direct inhibitor of eIF4F that targets its key enzymatic component, the DEAD-box RNA helicase eIF4A [41]. We have shown previously silvestrol is broadly potent against lymphoma cell lines [15]. To identify the relative importance of different PIM targets in the anti-lymphoma activities of PIM447, we tested it in combination with silvestrol. We hypothesized inhibition of translation activation by PIM447 upstream of where silvestrol acts would add little to silvestrol's potency, but if other key survival pathways are also inhibited then antitumor effects would be increased. As shown in Figure 3A, addition of PIM447 to silvestrol (48-hour exposure in contrast to initial PIM447 IC_50_ determinations at 72 hours) resulted in no further decrease in viability in ABC lines, regardless of baseline PIM447 sensitivity. These data argue interruption of translational activation is the key effect of PIM inhibition in sensitive cells. Results were similar for ibrutinib, adding little to the overall toxic effect of single-agent silvestrol at 48 hours (Figure S2).

To interrogate translation more directly, we assessed the effect of PIM447 on O-propargyl-puromycin (OPP) incorporation [42, 43] in comparison to silvestrol (Figure 3B). PIM447 led to strong decline of OPP incorporation, similar to silvestrol, in sensitive lines only. Similarly, PIM447 resulted in potent loss of translationally regulated proteins CyclinD3 and c-MYC only in sensitive lines (Figure 3C). These data suggest a requirement for PIM activity to maintain translational activation in sensitive lines that is not present in resistant lines.

### Clinical DLBCL PIM1 mutations largely preserve its pro-survival activities

Our data suggest an important role for PIM kinase activity in many cases of ABC-DLBCL. *PIM1*, however, is among the most frequently mutated genes in DLBCL clinical samples, with predominance in the ABC subtype (Table S1) [44–46]. While off targeting of activation–induced cytidine deaminase (AID) is well-established as a mechanism leading to mutations in *PIM1*, the functional significance of these mutations is unknown. We pooled mutations from published DLBCL datasets, revealing 92 instances of *PIM1* mutations resulting in alteration to the amino acid sequence (Table S1). Of these, 86 (94%) were missense, 3 (3%) were nonsense, and 3 (3%) were frame-shift (Figure 4A). This patterns suggests possible selection for mutations that at least preserve PIM1's functions, but because it is a constitutively active kinase when expressed, a majority of random missense mutations are likely to be damaging rather than activating. To focus functional analysis, we chose all amino-acid residues affected recurrently, illustrated in Figure 4B and listed in Table 1. Either the most common substitution or the one predicted by Polyphen2 to be most damaging at each recurrently mutated residue was generated by site-directed mutagenesis in an MSCV-based vector co-expressing GFP. Each was introduced retrovirally to FL5.12 cells to generate a starting point of ~20% GFP+, followed by IL3 withdrawal, then rescue after 24 hours (Figure 4C). As expected, wild-type *PIM1* enriches potently as a result of the selective pressure posed by IL3 withdrawal, while kinase-dead *PIM1* (K67M) does not. When 300 nM PIM447 was included during IL3 withdrawal and rescue, PIM1 showed no enrichment. This system therefore allows assessment of *PIM1* mutant alleles for ability to promote survival advantage during growth-factor withdrawal in comparison to wild-type and also test for ability to bypass PIM447. As shown in Figure 4D, nearly all recurrent DLBCL *PIM1* mutations preserved pro-survival function of the kinase, although some enriched less strongly than wild type, and one (L182F) failed to enrich at all, similar to kinase-dead. None of the mutations promoted resistance to PIM447.

**Table 1:** Recurrent clinical DLBCL somatic mutations in PIM1

Residue	Times found	Substitution	Enrich in 5.12s	Resistant to PIM447	AID hotspot
L2	4	F(3), V(1)	Yes	No	Yes, all
K24	4	N(4)	Yes	No	Yes
G28	4	D(4)	Yes	No	Yes
Q37	2	H(2)	Yes	No	Yes
S75	2	F(2)	Yes	No	Yes
E79	4	D(4)	Yes	No	Yes
P81	4	I(1), A(1), C(1), S(1)	Yes	No	Yes, all
S97	10	T(1), R(1), N(8)	Yes	No	Yes
P125	2	S(1), T(1)	Yes	No	Yes, all
E135	3	Q(1), K(2)	Yes	No	Neither
S146	3	N(1), R(2)	Yes	No	Yes, all
L164	4	V(1), F(3)	Yes	No	Yes, all
L182	2	F(2)	No	No	Yes
L184	3	V(1), F(2)	No	No	Yes, all
L193	2	F(2)	Yes	No	Yes

Amino acid altering mutations found at least two times in separated clinical samples, with their amino acid substitutions and the number of times that change was found in parenthesis. The results of enrichment assays in FL.512 cells after cytokine withdrawal and treatment with PIM447, and whether that mutation is in at an AID hotspot motif are also shown.

## DISCUSSION

To date, no PIM kinase inhibitor has progressed to regulatory approval. The PIM1-selective inhibitor SGI- 1776 entered phase 1 in 2008, but the trial ended early due to cardiac toxicity (NCT00848601). AZD1208 was developed as a pan-PIM inhibitor and was evaluated in a Phase 1 trail for solid tumors and lymphoma, with anti-tumor results not yet made public (NCT01588548). A planned trial for the drug in acute myeloid leukemia, where it had strong preclinical activity [19], was canceled (NCT01489722). Currently, PIM447 is the only anti-PIM kinase compound under clinical evaluation, being assessed in phase 1 in heavily pretreated multiple myeloma patients. Results presented at the 2014 American Society of Hematology Annual Meeting revealed maximum tolerated oral dose of 500 mg daily [47]. Dose-limiting toxicities included thrombocytopenia and fatigue. Of 48 patients evaluable for response, 10 (20.8%) had clinical benefit of molecular remission or better, and another 23 patients had stable disease, for an overall disease control rate of 68.8%.

In addition to off-target toxicity that led to the failure of SGI-1776, two other major issues have dogged development of PIM inhibition as a therapeutic approach. First, is the common expression and functional redundancy of the three family members, a problem that should be overcome by drugs like AZD1208 and PIM447, specifically designed as pan-PIM inhibitors. The second problem, however, is more persistent – defining patients in which PIM inhibition is likely to show significant antitumor activity. PIM synergizes potently with c-MYC and can promote tumorigenesis through multiple other targets but is nonetheless, by itself, considered weakly oncogenic [3]. For one thing, PIM has few if any unique substrates, overlapping in activity with multiple other signaling pathways that could be expected to provide readily available resistance pathways against pharmacologic PIM inhibition. Multiple myeloma has emerged as a promising area for PIM-inhibitor development associated with high activity of PIM2. For this reason, much of the ongoing and planned development for PIM447 is focused in this area. The potential of PIM inhibition in most other disease areas, however, remains unclear.

Here, we assessed PIM447 in a variety of non-myeloma lymphoid disease models. While selected lines from various diseases showed sensitivity, ABC-DLBCL emerged as the most sensitive group. This is perhaps not surprising since it the mostly biologically similar to myeloma, being derived from a B-cell population on its way to the terminal differentiation into plasma cells, the myeloma precursor population [48]. *PIM1* and *PIM2* are both defining features of the ABC gene-expression signature that permit distinguishing it from GCB, and both are turned on in expression by NF-kB, ABC's molecular hallmark. We find, however, that while PIM inhibition is potent against some ABC lines, others are completely resistant. The important distinguisher appears to be the manner in which signaling is turned on, with those lines activating NF-kB downstream from the BCR, thereby bypassing several redundant signaling pathways, being most likely to show sensitivity. Sensitive lines had PIM447-susceptible activation of cap-dependent translation, while resistant lines maintained translation regardless of PIM inhibition. The redundancy of signaling promoted by PIM activity is thus fully on display.

These findings suggest a possible role for PIM kinase inhibition in the therapy of ABC-DLBCL, which responds poorly to standard combination chemoimmunotherapy compared to GCB-DLBCL [49]. Current efforts to improve outcomes for ABC patients are focused on its key biologic differences with GCB. Lenalidomide, for example, can interrupt a positive-feedback signaling loop mediated by the transcription factor IRF4, which both helps maintain NF-kB signaling and inhibits an autocrine death pathway [50]. A recent single-arm phase 2 trial found lenalidomide combined with R-CHOP overcame the negative prognostic significance of non-GCB cases [51], and a randomized phase 3 follow-up is underway. Ibrutinib also shows promise for ABC-DLBCL but so far has been assessed only in heavily pre-treated relapsed/refractory patients [52]. Ibrutinib's target BTK sits downstream of BCR activation and upstream of the CBM complex. It therefore can disrupt the crucial activation of NF-kB that results from oncogenic BCR activity through mechanisms such as chronic-active signaling and stimulation by antigen [35]. For those cases with lesions that bypass the upper part of the signaling via CARD11 coiled-coil domain mutations or/or loss of A20, however, BTK inhibition may be less effective. The signaling is by no means linear, with feedback loops and additional input from MYD88-L265P/TLR signaling [48], as seen in our data with the line Ly10, sensitive to both PIM447 and ibrutinib. Clinically, however, evidence already has emerged of reduced chance of ibrutinib response when downstream lesions are present [52]. It is encouraging, therefore, that this set of cases, predicted to be left with unmet clinical need by BTK inhibitors, is the very set that may best respond to pan-PIM kinase inhibition, as suggested by our data.

Our data also reiterate cap-dependent translation initiation by eIF4F as a vital activity downstream of signaling and perhaps a better therapeutic target than the PIM kinases, BTK, or any other upstream messenger molecule. Silvestrol is significantly more potent than PIM447, ibrutinib, and most other signaling inhibitors highlighted by our data here, our previous work, and multiple other studies [15, 53, 54]. Silvestrol and other drugs targeting the eIF4A helicase, however, are not yet clinically available and to our knowledge none is on a development pathway putting it close to clinical evaluation in the near term. For that reason, studies such as the current report of clinically available compounds remain necessary and important to help identify patients more likely to benefit.

Finally, we undertake the first meta-analysis and functional evaluation of *PIM1* mutations found in DLBCL. Fifteen years ago, the gene was found among several prominent proto-oncogenes that are recurrently mutated by aberrant activity of the mutagenic enzyme AID [44], which has crucial roles in affinity maturation and class-switch recombination in non-malignant B-cells. Subsequent reports, including whole-exome and whole-genome sequencing, verify *PIM1* consistently as among the top three or four most frequently mutated genes in DLBCL clinical samples, especially ABC-derived cases [33, 45, 46]. The significance of these mutations, however, if any, is completely unknown. Data reported at the 2014 American Society of Hematology Annual Meeting but not yet published suggested a marginal negative prognostic significance for *PIM1* mutation-containing cases [55], but even this does little to clarify the situation. For example, such significance may not be due to altered activity of PIM1 itself – the *PIM1* mutations may merely be a marker for cases with the highest degree of aberrant AID activity that is the real driver of negative prognosis. This is supported by recent detailed mechanistic studies showing *PIM1*'s location in both a super-enhancer and at a site of frequent convergent transcription, a “perfect storm” for targeting by aberrant AID activity [56, 57]. Alternatively, *PIM1* mutations may be selected during disease development that enhance its oncogenic functions, in particular its well-described synergy with the common lymphoma oncogene c-MYC. PIM1, however, like all PIM family members is a constitutively active kinase that requires no post-translational modification for full activity. Any random mutation therefore is far more likely to be damaging than enhancing to such a kinase. Finally, a third and much less likely possibility is that ABC-DLBCL lymphomagenesis actually selects for PIM1 loss of function. Based on our data, we favor two conclusions: First, PIM1 mutations in DLBCL result overwhelmingly from aberrant AID activity and are driven primarily by this biology than by a process in which mutations to PIM1 are a primary or required tumorigenic process. For example, a great majority of the mutations overall (Table S1) and especially recurrent mutations (Table 1) are at clear AID hotspots. Second, the great majority of PIM1 mutations in DLBCL clinical samples preserve the protien's functions: only 6% of the mutations in our meta-analysis were either nonsense or frame-shift changes. Nearly all recurrent missense mutations, meanwhile, retained ability to rescue FL5.12 cells from IL3 withdrawal in a manner similar to the wild-type protein. There was no evidence of either enhancement of activity or of acquired ability to bypass pan-PIM inhibition with PIM447 (Figure 4C–4D). Therefore, while PIM1 activity may be dispensable to selected cases, overall preservation of PIM1 activity appears favored.

## MATERIALS AND METHODS

### Cell culture

All DLBCL cell lines were purchased from Deutsche Sammlung von Mikroorganismen und Zellkulturen (DSMZ), and verified by STR-fingerprinting on 11/2013. All lines tested negative for mycoplasma using the PlasmoTest Kit (Invivogen; REP-PT1). HBL1, TMD8, U2932, Riva, Toledo, OZ, SU-DHL-4, WSU-DLCL-2, MD901 lines were grown in RPMI culture media (Corning) with 10% fetal bovine serum (FBS, VWR) and penicillin/streptomycin (P/S), R10 media. OCI-Ly-2, OCI-Ly3, OCI-Ly-10 and OCI-Ly19 were grown in IDMEM in 20% FBS and P/S, I20 media. DB, SU-DHL-6, SU-DHL-10, Farage, Karpas-422, and SKI-DLCL-1 were grown in RPMI with 20% FBS and P/S, R20 media. The FL5.12 cells were a gift from the Wendel Lab, MSKCC, NY and grown in RPMI with 10% FBS, ± WeHi-3B supernatant and murine IL-3 (400 ρM, eBioscience). Cell lines were grown at 37 degrees Celsius in 5% CO_2_.

### Reagents

PIM447 and AEB071 were provided under material-transfer agreement (MTA) with Novartis Oncology. Ibrutinib was provided under MTA with Pharmacyclics. Silvestrol was a gift of Jerry Pelletier, McGill University. AZD1208 was purchased from Selleck Chemicals.

### Cell viability

Cells were seeded at 3,000 cells/100 μL in 96- well plates with serial dilutions of each inhibitor or the combination of Silvestrol and either PIM447 or ibrutinib. Viability was assessed using the Cell Titer Glo reagent (Promega) per the manufacturer's protocol at 72 hours, except in the case of Silvestrol and the Silvestrol/PIM447 combination experiments, which were assessed at 48 hours. Luminescence was detected on the BioTek Synergy HT plate reader. Viability data was used to determine IC_50_ using non-linear curve fit regression in GraphPad Prism 6.

### qRT PCR

Total RNA was isolated from cell lines using the RNeasy Mini Kit (Qiagen). cDNA was generated using the Taqman Reverse Transcriptase Kit (Roche). qRT-PCR was performed using an Applied Biosystems Prism 7000 Sequence Detection system with Taqman probes according to manufacturer specifications. Probes used were: Hs0165498_m1 (PIM1), Hs00179139_m1 (PIM2), Hs00420511_m1 (PIM3), and Hs02758991_g1 (GAPDH) for normalizing the data, using the 2^−ΔΔCT^.

### Western-blot analysis

Cells were treated as indicated and protein isolated using RIPA lysis buffer as described previously [58], with phosphatase inhibitors (Roche) and Protease Halt (Thermo) and Triton-X100. 30 μg of protein, quantified by BCA Assay (Thermo), was loaded onto 10% SDS-PAGE gel and resolved by electrophoresis, then transferred to PVDF membrane. Protein was detected using autoradiography film (GeneMate). Antibodies were diluted in 5% Milk in TBST and incubated overnight at 4 degrees Celsius. Cell Signaling Technologies 1:1000: PIM2, PIM3, Ribosomal S6 protein, pRibosomal S6 protein Ser^240/244^, 4EBP1, p4EBP-1 Ser^65^, AKT, pAKT Ser^473^, IκBα, pIκBα Ser^32^, CyclinD3, Myc, Tsc2, eIF4B, peIF4B Ser^422^, eIF4E, peIF4E Ser^209^. Santa-Cruz Biotechnologies 1:200: pTsc2 Ser^1798^, PIM1. Sigma 1:2000: α-Tubulin, HRP conjugated anti-rabbit and anti-mouse secondaries.

### FL5.12 PIM2-driven line

The Phoenix packaging cell line was seeded at 233,000 cells/mL for 16 hours. Then transfected with human PIM2 cDNA cloned in to the multiple cloning site of the p-MIG vector using X-treme GENE 9 DNA transfection reagent (Roche). The transfection cocktail consisted of 100 μL DMEM media, 1 μg DNA and 3 μL of X-treme GENE 9. Media on Phoenix cells was changed after 24 hours and at 48 hours collected and filter sterilized through a 0.45 μm filter. 100,000 FL5.12 cells were resuspended in 600 μL of viral supernatant and 120 μL of 5X infection solution was added (4 mL FL5.12 media, 1 mL WeHi supernatant, Polybreen and murine IL-3). The infection was repeated two more times, at least 6 hours apart to achieve maximum viral titer, by adding sterilized viral supernatant and 5X infection solution to the existing plate of cells. Six hours after the final infection cells were plated in fresh FL5.12 media and allowed to recover for 24 hours and initial infection was assessed by FACS using the Guava EasyCyte flow cytometer to check GFP levels. 500,000 cells were withdrawn from cytokine by washing 4 times and plating in cytokine-free media for 24 hours, or until majority of cells have shrunk in size and appear unhealthy, then put back in regular FL5.12 media until recovered healthy appearance then GFP was assessed by FACS. This process was repeated until cells reached > 95% GFP then they were gradually split into cytokine-free media until able to grow to confluence. FACS was used again to ensure cells were now 100% GFP positive.

### O-propargyl puromycin (OPP) incorporation

100,000 cells were seeded and treated with indicated inhibitor or vehicle. At 23 hours they were pulsed with 50 μM OPP (Jena Bioscience) for one hour as described previously [42]. Cells were then collected, washed once with PBS, fixed in 4% paraformaldehyde in PBS at 4 degrees Celsius for 15 minutes, and permeabilized with 3% BSA. 1% Saponin in PBS at room temperature for 15 minutes. CuAAC detection of OPP labeled proteins was then performed using the Click-iT Cell Reaction Buffer Kit according to manufacturer specifications with the Alexa Fluor 488 Azide (Thermo Fisher). After labeling GFP was assessed by FACS and analyzed using FlowJo software.

### PIM1 competition assay ± PIM447

Site-directed mutagenesis was used to create the PIM1 mutants from the short form of human PIM1 cDNA cloned in to the multiple cloning site of the pMIG vector according to the QuikChange II Site-Directed mutagenesis Kit manufacturers protocol (Aligent Technologies). The primers used are listed in Table S2. Mutants were confirmed by sanger sequencing and FL5.12 cells were infected with each mutant individually as described above in FL5.12 PIM2 driven line. After recovery from three infections initial GFP was assessed by FACS and then mixed with uninfected FL5.12 cells to create a stable mix of approximately 20% GFP positive cells. 500,000 cells were then washed out of IL-3 and simultaneously treated with either 300 nM PIM447 or vehicle. After 24 hours, cells were put back in normal FL5.12 media with either PIM447 or vehicle. Cells were allowed to recover and then GFP was assessed by FACS.

## SUPPLEMENTARY MATERIALS FIGURES AND TABLES





## References

[1] Warfel NA, Kraft AS PIM kinase (and Akt) biology and signaling in tumors. Pharmacol Ther.

[2] Mondello P, Cuzzocrea S, Mian M (2014). Pim kinases in hematological malignancies: where are we now and where are we going?. J Hematol OncolJ Hematol Oncol.

[3] Nawijn MC, Alendar A, Berns A (2010). For better or for worse: the role of Pim oncogenes in tumorigenesis. Nat Rev Cancer.

[4] Theo Cuypers H, Selten G, Quint W, Zijlstra M, Maandag ER, Boelens W, van Wezenbeek P, Melief C, Berns A (1984). Murine leukemia virus-induced T-cell lymphomagenesis: Integration of proviruses in a distinct chromosomal region. Cell.

[5] Breuer ML, Cuypers HT, Berns A (1989). Evidence for the involvement of pim-2, a new common proviral insertion site, in progression of lymphomas. EMBO J.

[6] Mikkers H, Allen J, Knipscheer P, Romeyn L, Hart A, Vink E, Berns A (2002). High-throughput retroviral tagging to identify components of specific signaling pathways in cancer. Nat Genet.

[7] Mikkers H, Nawijn M, Allen J, Brouwers C, Verhoeven E, Jonkers J, Berns A (2004). Mice deficient for all PIM kinases display reduced body size and impaired responses to hematopoietic growth factors. Mol Cell Biol.

[8] Domen J, van der Lugt NM, Acton D, Laird PW, Linders K, Berns A (1993). Pim-1 levels determine the size of early B lymphoid compartments in bone marrow. J Exp Med.

[9] Katakami N, Kaneto H, Hao H, Umayahara Y, Fujitani Y, Sakamoto K 'ya, Gorogawa S, Yasuda T, Kawamori D, Kajimoto Y (2004). Role of Pim-1 in Smooth Muscle Cell Proliferation. J Biol Chem.

[10] Muraski JA, Rota M, Misao Y, Fransioli J, Cottage C, Gude N, Esposito G, Delucchi F, Arcarese M, Alvarez R, Siddiqi S, Emmanuel GN, Wu W (2007). Pim-1 regulates cardiomyocyte survival downstream of Akt. Nat Med.

[11] Gapter LA, Magnuson NS, Ng K, Hosick HL (2006). Pim-1 kinase expression during murine mammary development. Biochem Biophys Res Commun.

[12] Fox CJ, Hammerman PS, Cinalli RM, Master SR, Chodosh LA, Thompson CB (2003). The serine/threonine kinase Pim-2 is a transcriptionally regulated apoptotic inhibitor. Genes Dev.

[13] Qian KC, Wang L, Hickey ER, Studts J, Barringer K, Peng C, Kronkaitis A, Li J, White A, Mische S, Farmer B (2005). Structural Basis of Constitutive Activity and a Unique Nucleotide Binding Mode of Human Pim-1 Kinase. J Biol Chem.

[14] Hsi ED, Jung S-H, Lai R, Johnson JL, Cook JR, Jones D, Devos S, Cheson BD, Damon LE, Said J (2008). Ki67 and PIM1 expression predict outcome in mantle cell lymphoma treated with high dose therapy, stem cell transplantation and rituximab: a Cancer and Leukemia Group B 59909 correlative science study. Leuk Lymphoma.

[15] Schatz JH, Oricchio E, Wolfe AL, Jiang M, Linkov I, Maragulia J, Shi W, Zhang Z, Rajasekhar VK, Pagano NC, Porco JA, Teruya-Feldstein J, Rosen N (2011). Targeting cap-dependent translation blocks converging survival signals by AKT and PIM kinases in lymphoma. J Exp Med.

[16] Alizadeh AA, Eisen MB, Davis RE, Ma C, Lossos IS, Rosenwald A, Boldrick JC, Sabet H, Tran T, Yu X, Powell JI, Yang L, Marti GE (2000). Distinct types of diffuse large B-cell lymphoma identified by gene expression profiling. Nature.

[17] Wright G, Tan B, Rosenwald A, Hurt EH, Wiestner A, Staudt LM (2003). A gene expression-based method to diagnose clinically distinct subgroups of diffuse large B cell lymphoma. Proc Natl Acad Sci USA.

[18] Tamburini J, Green AS, Bardet V, Chapuis N, Park S, Willems L, Uzunov M, Ifrah N, Dreyfus F, Lacombe C, Mayeux P, Bouscary D (2009). Protein synthesis is resistant to rapamycin and constitutes a promising therapeutic target in acute myeloid leukemia. Blood.

[19] Keeton EK, McEachern K, Dillman KS, Palakurthi S, Cao Y, Grondine MR, Kaur S, Wang S, Chen Y, Wu A, Shen M, Gibbons FD, Lamb ML (2013). AZD1208, a potent and selective pan-pim kinase inhibitor, demonstrates efficacy in preclinical models of acute myeloid leukemia. Blood.

[20] Lu J, Zavorotinskaya T, Dai Y, Niu X-H, Castillo J, Sim J, Yu J, Wang Y, Langowski JL, Holash J, Shannon K, Garcia PD (2013). Pim2 is required for maintaining multiple myeloma cell growth through modulating TSC2 phosphorylation. Blood.

[21] Asano J, Nakano A, Oda A, Amou H, Hiasa M, Takeuchi K, Miki H, Nakamura S, Harada T, Fujii S, Kagawa K, Endo I, Yata K (2011). The serine/threonine kinase Pim-2 is a novel anti-apoptotic mediator in myeloma cells. Leukemia.

[22] Hiasa M, Teramachi J, Oda A, Amachi R, Harada T, Nakamura S, Miki H, Fujii S, Kagawa K, Watanabe K, Endo I, Kuroda Y, Yoneda T (2014). Pim-2 kinase is an important target of treatment for tumor progression and bone loss in myeloma. Leukemia.

[23] Chen WW (2005). Pim Family Kinases Enhance Tumor Growth of Prostate Cancer Cells. Mol Cancer Res.

[24] Fujii C, Nakamoto Y, Lu P, Tsuneyama K, Popivanova BK, Kaneko S, Mukaida N (2005). Aberrant expression of serine/threonine kinase Pim-3 in hepatocellular carcinoma development and its role in the proliferation of human hepatoma cell lines. Int J Cancer.

[25] Popivanova BK, Li Y-Y, Zheng H, Omura K, Fujii C, Tsuneyama K, Mukaida N (2007). Proto-oncogene, Pim-3 with serine/threonine kinase activity, is aberrantly expressed in human colon cancer cells and can prevent Bad-mediated apoptosis. Cancer Sci.

[26] Bullock AN, Debreczeni J, Amos AL, Knapp S, Turk BE (2005). Structure and substrate specificity of the Pim-1 kinase. J Biol Chem.

[27] Kumar A, Mandiyan V, Suzuki Y, Zhang C, Rice J, Tsai J, Artis DR, Ibrahim P, Bremer R (2005). Crystal structures of proto-oncogene kinase Pim1: a target of aberrant somatic hypermutations in diffuse large cell lymphoma. J Mol Biol.

[28] Chen LS, Redkar S, Bearss D, Wierda WG, Gandhi V (2009). Pim kinase inhibitor, SGI-1776, induces apoptosis in chronic lymphocytic leukemia cells. Blood.

[29] Garcia PD, Langowski JL, Wang Y, Chen MY, Castillo J, Fanton C, Ison M, Zavorotinskaya T, Dai Y, Lu J, Niu X-H, Basham S, Chan J (2014). Pan-PIM Kinase Inhibition Provides a Novel Therapy for Treating Hematological Cancers. lin Cancer Res.

[30] Zhu N, Ramirez LM, Lee RL, Magnuson NS, Bishop GA, Gold MR (2002). CD40 Signaling in B Cells Regulates the Expression of the Pim-1 Kinase Via the NF-κB Pathway. J Immunol.

[31] Li J, Peet GW, Balzarano D, Li X, Massa P, Barton RW, Marcu KB (2001). Novel NEMO/IκB Kinase and NF-κB Target Genes at the Pre-B to Immature B Cell Transition. J Biol Chem.

[32] Lenz G, Davis RE, Ngo VN, Lam L, George TC, Wright GW, Dave SS, Zhao H, Xu W, Rosenwald A, Ott G, Muller-Hermelink HK, Gascoyne RD (2008). Oncogenic CARD11 mutations in human diffuse large B cell lymphoma. Science.

[33] Zhang J, Grubor V, Love CL, Banerjee A, Richards KL, Mieczkowski PA, Dunphy C, Choi W, Au W-Y, Srivastava G, Lugar PL, Rizzieri DA, Lagoo AS (2013). Genetic heterogeneity of diffuse large B-cell lymphoma. Proc Natl Acad Sci.

[34] Compagno M, Lim WK, Grunn A, Nandula SV, Brahmachary M, Shen Q, Bertoni F, Ponzoni M, Scandurra M, Califano A, Bhagat G, Chadburn A, Dalla-Favera R (2009). Mutations of multiple genes cause deregulation of NF-κB in diffuse large B-cell lymphoma. Nature.

[35] Davis RE, Ngo VN, Lenz G, Tolar P, Young RM, Romesser PB, Kohlhammer H, Lamy L, Zhao H, Yang Y, Xu W, Shaffer AL, Wright G (2010). Chronic active B-cell-receptor signalling in diffuse large B-cell lymphoma. Nature.

[36] Ngo VN, Young RM, Schmitz R, Jhavar S, Xiao W, Lim K-H, Kohlhammer H, Xu W, Yang Y, Zhao H, Shaffer AL, Romesser P, Wright G (2011). Oncogenically active MYD88 mutations in human lymphoma. Nature.

[37] Yang J, Wang J, Chen K, Guo G, Xi R, Rothman PB, Whitten D, Zhang L, Huang S, Chen J-L (2013). eIF4B phosphorylation by Pim kinases plays a critical role in cellular transformation by Abl oncogenes. Cancer Res.

[38] Landon AL, Muniandy PA, Shetty AC, Lehrmann E, Volpon L, Houng S, Zhang Y, Dai B, Peroutka R, Mazan-Mamczarz K, Steinhardt J, Mahurkar A, Becker KG (2014). MNKs act as a regulatory switch for eIF4E1 and eIF4E3 driven mRNA translation in DLBCL. Nat Commun.

[39] Ma J, Arnold HK, Lilly MB, Sears RC, Kraft A (2007). Negative regulation of Pim-1 protein kinase levels by the B56β subunit of PP2A. Oncogene.

[40] Woodland RT, Fox CJ, Schmidt MR, Hammerman PS, Opferman JT, Korsmeyer SJ, Hilbert DM, Thompson CB (2008). Multiple signaling pathways promote B lymphocyte stimulator dependent B-cell growth and survival. Blood.

[41] Cencic R, Carrier M, Galicia-Vázquez G, Bordeleau M-E, Sukarieh R, Bourdeau A, Brem B, Teodoro JG, Greger H, Tremblay ML, Porco JA, Pelletier J (2009). Antitumor Activity and Mechanism of Action of the Cyclopenta[b]benzofuran, Silvestrol Preiss T, editor. PLoS ONE.

[42] Liu J, Xu Y, Stoleru D, Salic A (2012). Imaging protein synthesis in cells and tissues with an alkyne analog of puromycin. Proc Natl Acad Sci.

[43] Signer RAJ, Magee JA, Salic A, Morrison SJ (2014). Haematopoietic stem cells require a highly regulated protein synthesis rate. Nature.

[44] Pasqualucci L, Neumeister P, Goossens T, Nanjangud G, Chaganti RS, Kuppers R, Dalla-Favera R (2001). Hypermutation of multiple proto-oncogenes in B-cell diffuse large-cell lymphomas. Nature.

[45] Morin RD, Mendez-Lago M, Mungall AJ, Goya R, Mungall KL, Corbett RD, Johnson NA, Severson TM, Chiu R, Field M, Jackman S, Krzywinski M, Scott DW (2011). Frequent mutation of histone-modifying genes in non-Hodgkin lymphoma. Nature.

[46] Lohr JG, Stojanov P, Lawrence MS, Auclair D, Chapuy B, Sougnez C, Cruz-Gordillo P, Knoechel B, Asmann YW, Slager SL, Novak AJ, Dogan A, Ansell SM (2012). Discovery and prioritization of somatic mutations in diffuse large B-cell lymphoma (DLBCL) by whole-exome sequencing. Proc Natl Acad Sci.

[47] Raab M, Ocio E, Thomas S, Gunther A, Goh Y-T, Lebovic D, Jakubowiak AJ, Song D, Ziang F, Patel A, Vanasse KG, Kumar S Phase 1 Study Update of the Novel Pan-Pim Kinase Inhibitor LGH447 in Patients with Relapsed/Refractory Multiple Myeloma. https://ashconfexcom/ash/2014/webprogram/Paper69283html.

[48] Shaffer AL, Young RM, Staudt LM (2012). Pathogenesis of Human B Cell Lymphomas. Annu Rev Immunol.

[49] Roschewski M, Staudt LM, Wilson WH (2014). Diffuse large B-cell lymphoma-treatment approaches in the molecular era. Nat Rev Clin Oncol.

[50] Yang Y, Shaffer I Arthur L, Emre NCT, Ceribelli M, Zhang M, Wright G, Xiao W, Powell J, Platig J, Kohlhammer H, Young RM, Zhao H, Yang Y (2012). Exploiting Synthetic Lethality for the Therapy of ABC Diffuse Large B Cell Lymphoma. Cancer Cell.

[51] Nowakowski GS, LaPlant B, Macon WR, Reeder CB, Foran JM, Nelson GD, Thompson CA, Rivera CE, Inwards DJ, Micallef IN, Johnston PB, Porrata LF, Ansell SM (2014). Lenalidomide Combined With R-CHOP Overcomes Negative Prognostic Impact of Non–Germinal Center B-Cell Phenotype in Newly Diagnosed Diffuse Large B-Cell Lymphoma: A Phase II Study. J Clin Oncol.

[52] Wilson WH, Young RM, Schmitz R, Yang Y, Pittaluga S, Wright G, Lih C-J, Williams PM, Shaffer AL, Gerecitano J, de Vos S, Goy A, Kenkre VP (2015). Targeting B cell receptor signaling with ibrutinib in diffuse large B cell lymphoma. Nat Med.

[53] Boussemart L, Malka-Mahieu H, Girault I, Allard D, Hemmingsson O, Tomasic G, Thomas M, Basmadjian C, Ribeiro N, Thuaud F, Mateus C, Routier E, Kamsu-Kom N (2014). eIF4F is a nexus of resistance to anti-BRAF and anti-MEK cancer therapies. Nature.

[54] Wolfe AL, Singh K, Zhong Y, Drewe P, Rajasekhar VK, Sanghvi VR, Mavrakis KJ, Jiang M, Roderick JE, Van der Meulen J, Schatz JH, Rodrigo CM, Zhao C (2014). RNA G-quadruplexes cause eIF4A-dependent oncogene translation in cancer. Nature.

[55] Ennishi D, Hoffer C, Shulha H, Mottok A, Farinha P, Chan F, Meissner B, Boyle M, Ben-Neriah S, Morin R, Marra M, Savage K, Sehn L Clinical Significance of Genetic Aberrations in Diffuse Large B Cell Lymphoma. https://ashconfexcom/ash/2014/webprogram/Paper70890html.

[56] Meng F-L, Du Z, Federation A, Hu J, Wang Q, Kieffer-Kwon K-R, Meyers RM, Amor C, Wasserman CR, Neuberg D, Casellas R, Nussenzweig MC, Bradner JE (2014). Convergent Transcription at Intragenic Super-Enhancers Targets AID-Initiated Genomic Instability. Cell.

[57] Qian J, Wang Q, Dose M, Pruett N, Kieffer-Kwon K-R, Resch W, Liang G, Tang Z, Mathé E, Benner C, Dubois W, Nelson S, Vian L (2014). B Cell Super-Enhancers and Regulatory Clusters Recruit AID Tumorigenic Activity. Cell.

[58] Amin AD, Rajan SS, Liang WS, Pongtornpipat P, Groysman MJ, Tapia EO, Peters TL, Cuyugan L, Adkins J, Rimsza LM, Lussier YA, Puvvada SD, Schatz JH (2015). Evidence Suggesting That Discontinuous Dosing of ALK Kinase Inhibitors May Prolong Control of ALK+ Tumors. Cancer Res.

