# Supplementary material for: Control of translational activation by PIM kinase in activated B-cell diffuse large B-cell lymphoma confers sensitivity to inhibition by PIM447
Source: Oncotarget. 2016 Aug 20;7(39):63362–73. doi: 10.18632/oncotarget.11457 (PMC5325370; doi:10.18632/oncotarget.11457)
Supplement: Supplementary file 1 [file oncotarget-07-63362-s001.pdf]

Control of translational activation by PIM kinase in activated B-cell diffuse large B-cell lymphoma confers sensitivity to inhibition by PIM447

Supplementary Materials

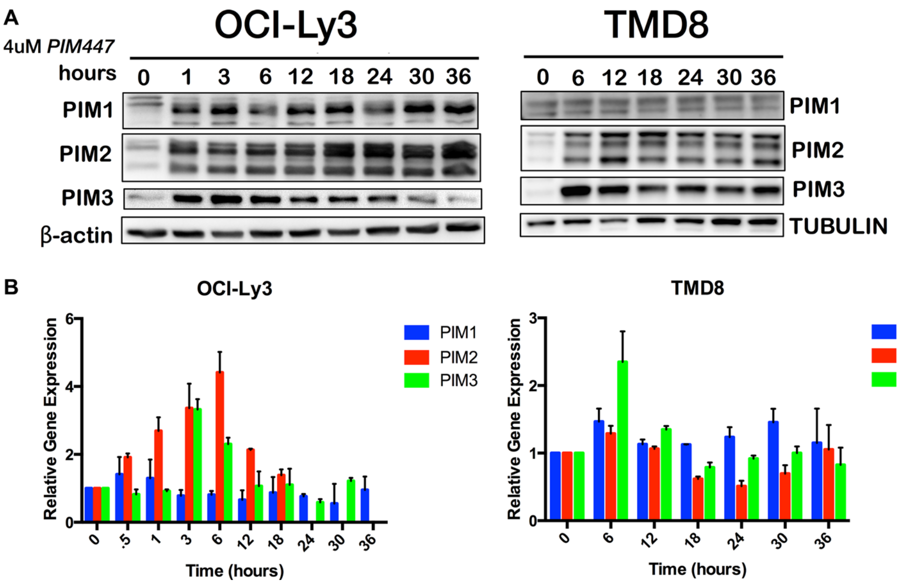

Supplementary Figure S1: Protein, but not mRNA levels, of all three PIM kinases increases with PIM447 treatment. (**A**) 5,000,000 cells were treated with 4 µM PIM447 for indicated time points. Cells were collected and protein isolated using RIPA lysis buffer, with protease and phosphatase inhibitors. 30 µg of protein was loaded onto a 10% polyacrylamide gel and transfered to a PVDF membrane and probed for the indicated proteins overnight at 4 degrees Celsius. (**B**) mRNA expression of human PIM kinases after PIM447 treatment. qRt-PCR was performed using probes for human PIM1, 2 and 3 on cDNA isolated from each cell line. 2-ΔΔCT normalized to GAPDH.

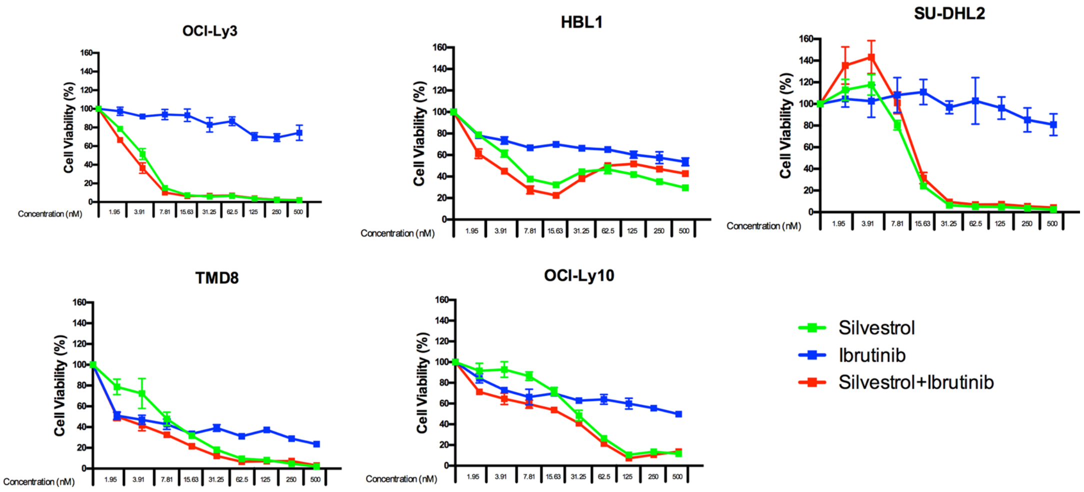

Supplementary Figure S2: Cells were seeded at 3,000 cells/100 µL in 96-well plates with serial dilutions of either Silverstrol, ibrutinib, or the combination. Final concentrations of both drugs alone were 1 uM, and viability was assessed using the Cell Tilter Glo reagent (Promega) per the manufacture's protocol at 48 hours. Luminescence was detected on the BioTek Synergy HT plate reader. Viability data was used to determine IC50 using non-linear curve fit regression in Graphpad Prism 6.

Supplementary Table S1: Meta analysis of somatic mutations in PIM1 from DLBCL clinical samples. See Supplementary_Table_S1

Supplementary Table S2: PCR primers used for site-directed mutagenesis of PIM1

Residue	Forward Primer	Reverse Primer
L2F	ctacgctggaagcttgatgttcttgtccaaaatcaactc	gagttgattttggacaagaacatcaagcttccagcgtag
K24N	gggcgccagattggtggcgtgcaggt	acctgcacgccaccaatctggcgccc
G28D	ctccttctccttgtcgggcgccagctt	aagctggcgcccgacaaggagaaggag
Q37H	ggcccacctggtaatgcgactccaggg	ccctggagtcgcattaccaggtgggcc
S75F	gtggagaaggaccggattttcgactgggga	tccccagtcgaaaatccggtccttctccac
E79D	gtgccattaggcagatctccccagtcgga	tccgactggggagatctgcctaatggcac
P81I	cactcgagtgccattaatcagctctccccagtcg	cgactggggagagctgattaatggcactcgagtg
S97N	tgctgaagaaggtgaactcgggtttctccgg	ccggagaaacccgagttcaccttcttcagca
P125S	ggagaggcccgagtcggtgcaagatct	agatcttgcaccgactcgggcctctcc
E135K	ttcgacttcatcacgaaaaggggagccctgc	gcagggctccccttttcgtgatgaagtcgaa
S146R	ctgccagaagaacctgcgggccagc	gctggcccgcaggttcttctggcag
L164F	ca a ctgcggggtgttcca ccgcgacat	atgtcgcggtggaacaccccgcagttg
E181D	gtcgatgagcttgagatcgccgcgattgagg	cctcaatcgcggcgatctcaagctcatcgac
L182F	agtcgatgagcttgaactcgccgcgattgag	ctcaatcgcggcgagttcaagctcatcgact
L184F	gcggcgagctcaagttcatcgacttcggg	cccga a gtcga tga a cttgagctcgccgc
L193F	tcgggggcgctgttcaaggacaccg	cggtgtccttgaacagcgcccccga
